# Tricuspid Valve Reconstruction in Patients with Right Heart Decompensation Due to Severe Tricuspid Regurgitation on LVAD Support

**DOI:** 10.3390/jcm13226705

**Published:** 2024-11-08

**Authors:** Henryk Welp, Jürgen Sindermann, Mirela Scherer

**Affiliations:** 1 Interdisciplinary Heart Failure Section, University Hospital Muenster, Albert Schweitzer Campus 1, A1, 48149 Muenster, Germany; juergen.sindermann@ukmuenster.de; 2 Department of Cardiothoracic Surgery, University Hospital Muenster, Albert Schweitzer Campus 1, A1, 48149 Muenster, Germany; mirela.scherer@ukmuenster.de; 3 Department of Cardiology I—Coronary and Peripheral Vascular Disease, Heart Failure, University Hospital Muenster, Albert Schweitzer Campus 1, A1, 48149 Muenster, Germany

**Keywords:** LVAD, right heart failure, tricuspid valve reconstruction, tricuspid valve insufficiency

## Abstract

**Background/Objectives**: Right ventricular (RV) dysfunction after left ventricular assist device (LVAD) implantation is associated with reduced survival and affects duration of hospitalization. Some patients with RV dysfunction on LVAD have significant tricuspid valve regurgitation (TR) with concomitant signs of RV failure. In these cases, tricuspid valve repair (TVR) may minimize clinical signs of RV failure. **Methods**: We report on two patients (one female, one male) developing significant TR receiving TVR through a right thoracotomy on LVAD support. **Results**: The time between LVAD implantation and TVR was 4 months and 50 months, respectively. The female patient could be discharged from hospital without TR and any signs of RV dysfunction. The male patient died 13 days later due to septic shock following mesenteric ischemia. At this time, echocardiography showed a competent tricuspid valve. **Conclusions**: In conclusion, these results provide insight into the clinical judgment of when TVR should be attempted. They suggest whether TVR may be a strategy to avoid hospitalization, minimize the clinical signs of RV insufficiency and improve quality of life in patients on LVAD support with severe TR and clinical signs of right heart dysfunction.

## 1. Introduction

Preoperative RV dysfunction is associated with progression after LVAD implantation in 20% of patients. This is associated with a high rate of morbidity and mortality [1,2,3]. TR is a common finding in patients with heart failure. It occurs secondary to RV dilatation with leaflet tethering and tricuspid annulus dilatation [4]. The significance of TR as an important factor in RV dysfunction in LVAD patients is controversial [1,5].

Some investigators reported that TR severity tended to decrease after LVAD implantation as left ventricular loading improved, suggesting that a TR severity of moderate or less would not need to be corrected [6]. Other single-center investigators have shown that the presence of moderate or severe TR at the time of LVAD implantation predicts an increased risk of subsequent RV failure [1,7]. Because the indication for surgical correction of TR remains uncertain, some investigators have adopted a more aggressive approach in favor of tricuspid valve annuloplasty in these patients [8,9,10].

Because annular dilatation is an underlying mechanism of nonorganic TR, three additional important factors are important in determining their occurrence: preload, afterload, and RV function. This may explain why TR is actually difficult to assess, as these factors may interfere with the severity of TR under different conditions.

Significant TR may not be detected echocardiographically despite significant valve pathology. However, this may indicate subsequent progression of TR. Patients with RV dysfunction and tricuspid annular dilatation, but without significant TR at the time of LVAD implantation, have often developed significant TR with LVAD support. Elimination of TR may improve RV function, resulting in reduced venous congestion with improved hepatic and renal perfusion.

We report our experience with two patients with significant TR and clinical signs of right heart failure while on LVAD support receiving TVR through a right thoracotomy.

## 2. Surgical Technique

The right femoral artery was cannulated with a 16 to 18 French arterial cannula, and the left femoral vein was cannulated with a 19 French venous cannula. The venous cannula was placed to the top of the inferior cava vein under echocardiographic control and cardiopulmonary bypass was started with 3 L/min. to 4 L/min. followed by LVAD flow reduction to 1 L/min.

A 7 to 8 cm skin incision was made in the right fifth intercostal space medial to the anterior axillary line. Under single lung ventilation, the inferior and superior cava veins were cross clamped and the right atrium was opened. When the tricuspid valve had been exposed, inspection revealed the annular dilatation. The ring sutures, 2-0 Ethibond Excel sutures (Ethicon Endo-Surgery, LLC, Cincinnati, OH, USA), were placed on the annulus, running from the anteroseptal commissure to the middle of the septal leaflet along the anterior and posterior leaflets. A Carpentier-Edwards Classic rigid ring (32 and 34 mm, respectively) was tied down with the sutures and placed on the annulus on a pump beating heart. The right atrium was then closed with a 4-0 Prolene suture in two-layer closure. Patients were weaned from cardiopulmonary bypass after double lung ventilation was reestablished and the LVAD flow was increased to the preoperative values.

### 2.1. Case 1

A 23-year-old female patient with the diagnosis of cutaneous T-cell lymphoma developed acute heart failure after anthracycline chemotherapy. Transesophageal echocardiography showed severely impaired biventricular function with an ejection fraction under 10%. Annular dilatation to 41.2 mm without significant TR was detected. Despite optimal medical therapy, heart function did not recover, leading to LVAD (Heart Ware) implantation. Postoperative severe right heart dysfunction was treated by establishing a temporary extracorporeal right heart support (RVAD). Subsequently, the patient recovered slowly from treatment and the RVAD could be removed 19 days later. At this time, the echocardiography revealed a better RV function and minimal TR.

About four months later, the patient showed clinical signs of right heart decompensation with ascites, pleural effusion, anasarca and renal insufficiency. Echocardiography showed an impaired RV function and high-grade TR. The tricuspid annulus was dilated to 42.2 mm. Clinical status did not improve and she also developed acute kidney failure requiring dialysis. Thereafter, we performed TVR as describe above. The patient recovered slowly after surgical procedure and was discharged home with a competent tricuspid valve, without ascites, anasarca and pleural effusion with improved kidney function without dialysis. Unfortunately, the patient died after a total support period of 582 days due to a graft vs. host reaction of the transplanted bone marrow.

### 2.2. Case 2

A 57-year-old male patient suffering from ischemic cardiomyopathy was admitted to our clinic in cardiogenic shock. To stabilize hemodynamics and improve organ function, an extracorporeal life support system was implanted. Transesophageal echocardiography showed severely impaired biventricular function with an ejection fraction under 20%. An annular dilatation to 46.1 mm without significant TR was detected. Since there was no recovery of heart function, an LVAD (Heart Ware) was implanted. Because of severe RV dysfunction, temporary extracorporeal right heart support was also established. Postoperatively, the patient recovered slowly and the RVAD could be removed 15 days later. At this time, the echocardiography revealed better right heart function and minimal TR.

The patient was stable for more than two years until he developed increasing signs of right heart failure. Echocardiography revealed an increasing tricuspid regurgitation. In light of this, the patient recurrently presented with deterioration in his clinical conditions. More than 3 years after LVAD implantation, the patient presented massive ascites and pleural effusion. Echocardiography showed an impaired RV function with severe tricuspid regurgitation and annular dilatation to 48.0 mm. Clinically, this was mirrored by increasing dyspnea, which led to admission of the patient for surgical reconstruction of the tricuspid valve. This was successfully performed and the patient initially recovered well. Postoperatively, the tricuspid valve showed only minimal insufficiency. However, during further treatment in the intensive care unit, the patient developed a paralytic ileus requiring surgical intervention (ileostomy). Unfortunately, subsequent complications based on paralytic ileus lead to septic shock, resulting in the patient’s death.

## 3. Discussion

Most patients undergoing LVAD implantation for left heart failure suffer from additional right heart dysfunction. Preoperative RV dysfunction is associated with progression and subsequent RV failure after surgery, determining high rates of morbidity and mortality. TR is generally common in this patient population with a preoperative incidence of nearly 50% [9]. However, preoperatively, TR has been associated with longer post–LVAD inotropic support and hospital stay as well as a trend toward higher mortality [7]. Some investigators reported that after LVAD implantation, the decrease in left atrial pressure and pulmonary congestion decreases RV afterload and improves tricuspid insufficiency. However, evidence that simply reducing RV afterload resolves TR is scarce [11,12]. Additionally, increased flow from the LVAD can lead to increased preload returning to the right ventricle, changing RV geometry, and thus exacerbate tricuspid insufficiency and RV failure [13]. The change in RV geometry is mainly a result from leftward shift of the interventricular septum. This shift can further impair RV function by decreasing the effective contribution of the septum to RV contraction and exacerbating TR through leaflet tethering

Given the risk associated with TR and the critical importance of RV function after LVAD implantation, TVR is often considered at the same time as LVAD implantation. In addition, TVR at the time of LVAD implantation appears to be associated with better survival [13,14,15].

Since many patients have moderate to less severe TR, and importantly the actual assessment of TR severity can vary over time, tricuspid annular dilatation seems to be a more reliable indicator of tricuspid valve pathology compared with TR.

During the postoperative period on LVAD support, in both patients we could observe an increase in TR from minimal to severe and an increase in the tricuspid annulus from 41.2 to 42.2 mm and from 46.1 to 48.0 mm, respectively. Further, the RV function deteriorated and venous congestion with decline in hepatic and renal function occurred. The patients also suffered from peripheral edema, ascites and pleural effusion. The tricuspid annulus is a component of both the tricuspid valve and the right ventricle. Thus, for TR to develop, both the tricuspid annulus and the right ventricle must dilate, if the morphology of the valve leaflets is preserved.

If severe TR is not diagnosed, the presence of annular dilatation is often not recognized. However, the assessment of the tricuspid valve at a given time by echocardiography is highly dependent on the patient’s preload and afterload. Since conditions may change from time to time, the absence of TR or the presence of only mild TR does not necessarily mean that the tricuspid valve annulus is free of abnormalities. Significant dilatation of the annulus does not always result in severe TR [4].

Once the annulus is dilated, its size may not spontaneously return to normal and may continue to dilate further. Therefore, more liberal indications for TVR (moderate or severe TR or tricuspid annulus > 40 mm) were applied [5]. In addition, it has been reported that tricuspid annular dilatation, even in the absence of a severe TR, has a negative impact on the mid-term survival after LVAD implantation (cut-off 43 mm) [15].

Delays in surgical intervention for severe tricuspid regurgitation (TR) in combination with left-sided heart valve surgery are associated with higher operative mortality. In this context, one study found that a longer latency between diagnosis and surgery correlates with increased mortality, especially in patients with massive preoperative TR and older age. From these data, it can be conditionally concluded that a delay in TVR in the context of LVAD implantation could also have a negative impact on long-term survival. Older patients and patients with severe TR appear to be particularly affected by this [16,17].

The essential message of our report is that in patients with severe TR and clinical signs of RV dysfunction while on LVAD support, tricuspid valve repair may be an option to minimize clinical signs of right heart insufficiency and improve quality of life. TR in LVAD patients arises from a combination of altered right ventricular geometry, annular dilatation, and the effects of continuous-flow devices on hemodynamics. Understanding these mechanisms is crucial for optimizing patient outcomes and guiding surgical decisions related to tricuspid valve management during LVAD implantation. Further research is needed to clarify the long-term implications of these interventions and improve strategies for managing TR in this population.

## Data Availability

Does not apply to this study.
